# Chicago Pathological Society

**Published:** 1884-11

**Authors:** 


CHICAGO PATHOLOGICAL SOCIETY. *Feb. 11, 1884. Regular Meeting.*

The Society was called to order by the President, Dr. Angear.

The minutes of the two last meetings were read and approved.

Dr. J. D. Skeer was unanimously elected to membership.

The names of Dr. Sarah Millen, West Liberty, Iowa, and Dr. McCullough, of Randolph and Halsted streets, were proposed by Drs. Angear and Tebbetts.

Dr. E. L. Holmes was elected Censor, to serve the remainder of the year, vice Dr. Walker, resigned.

The Society then being in readiness for work, Dr. E. L. Holmes presented a specimen of melanotic sarcoma of the eye. Also a large melanotic sarcoma of the upper retro-tarsal fold of the left conjunctiva.

The first had been diagnosticated by an opthalmoscopic examination of the right eye of Mr. ——, forty-one years of age, who was in excellent health, and presented no external evidence of the slightest disease of the eye. At the outer and lower part of the globe was a dark mass, beneath the retina, extending into the vitreous.

The patient had discovered a loss of sight in a part of his field of vision about six weeks before this examination, which was made late in November. Seven weeks after this, the second stages in the development of such tumors had supervened. These were characterized by pain, redness of the conjunctiva, increased hardness of the globe, further detachment of the retina, and dilatation of the pupil.

Enucleation of the globe, which had been advised at the first, was no longer rejected by the patient.

Bisection of the globe revealed the base of the tumor, extending from near the ciliary region in front to within a quarter of an inch of the optic disc. Upon this base was situated a smaller but higher portion with abrupt walls, which projected half an inch toward the center of the globe; the remaining portion of the eye was filled with a thin dark brown fluid, and a very small remnant of vitreous humor.

These tumors are usually composed of small round or spindle cells, and are quite malignant. The average duration of life is said to be four years. The recurrence of the growth is more frequent in the liver, or other internal organs, than in the orbit. Dr. Holmes stated that he had known several patients who had lived in apparent good health longer than this period, after the enucleation.

Mr. T., a German, 66 years of age, recently consulted Dr. Holmes for an inflammation of the left eye. A careless examination and a careless eversion of the upper lid simply revealed a chronic conjunctivitis, with considerable roughness ; this had existed a year.

A swelling of the upper lid had been observed during the last three months. By pressure on the upper portion of the lid, a large, black pedunculated tumor, three quarters of an inch in diameter, and a quarter of an inch thick, was made to protrude between the everted upper lid and globe.

By means of a ligature around the base of this growth, it could be drawn down in such a manner that its attachment to the conjunctiva at the retro-tarsal fold could be easily examined.

The conjunctiva thus tied and put upon the stretch by the ligature was readily divided so far from the tumor as to ensure apparently complete removal of all diseased tissue. The specimen shows a well defined tumor at its base, resting in the conjunctiva, without the slightest mutilation by the operation. Each tumor is rich in dark brown pigment Neither patient has a history of heredity.

The Society then proceeded to the discussion of "Therapeutics of Malarial Diseases."

Dr. E. L. Holmes opened the discussion by sketching the history of an obscure case of eye-trouble, caused by malaria, and cured by five-grain doses of quinine three times a day for two days.

Cases of tinnitus aurium had experienced no relief until large doses of quinine had been given.

Iritis simulates malarial eye trouble, but quinine, though often given, does no good.

The peculiarity about the pain in iritis is to come on about four in the morning. In all painful affections of the eye, where other remedies do but little good, give quinine.

Dr. Avery, continuing the discussion, stated that in certain severe cases of conjunctivitis, when applications had not relieved, quinine would both relieve and cure.

Dr. Haven had employed quinine as a sheet-anchor. With insane patients in the Wisconsin district, where he once resided, an attack of malaria seemed to hasten an attack of periodical insanity, and curing malarial complications relieved mental difficulty. Combined Fowler's solution with quinine, also a cold bath to reduce the temperature.

Dr. A. Lagorio considered quinine the chief remedy; many try substitutes, but return to the former,

An Italian physician some time ago advocated lemon-juice in decoction. Dr. L. had tried it faithfully and found it influenced the disease, but has had to return to quinine to complete a cure.

In Italy, also, electricity is much in vogue; considered it good when combined with tonics and quinine. Oil of eucalyptus and piperin, they use these also. Cures frequently. The eucalyptus tree, planted in the Roman Campagna, has changed an entire district from a deadly, uninhabitable tract in 1868 to a healthy and valuable locality. There is less fever there than is commonly found in Italy. Dr. L. had used eucalyptus oil between the attacks. In a case of a man with a very large spleen, had given quinine and carbonate iron; spleen normal in two months.

Dr. Bucher stated that in four of cases pernicious remittent fever, in which quinine seemed perfectly useless, the hot pack alone had relieved.

In a case of asthma, in which nothing relieved, he gave five grains of quinine, with the patient easy in ten minutes, and subsequent attacks were always thus relieved.

Dextro-quinine in double doses produces no ringing and other bad effects.

Dr. E. H. Root confirmed the efficacy of the hot water pack in malarial disease. The president, Dr. Angear, in a few remarks as to the real nature of malarial disease, whether of nervous origin or caused by bacteria, etc., quoted Watson's early edition, in the matter of deception of the patient, as to time of day when expecting at a certain hour a chill, and his consequent cure without medication.

The discussion being brought to its final conclusion by the lateness of the hour, the society, on motion, adjourned.

J. H. TIBBETTS, *Secretary*.

Park Institute, Feb. 11, 1884.

---

March 10, 1884. Regular meeting.

The society was called to order by Dr. Avery, the president, Dr. Angear, being absent.

The minutes of the last meeting were read and approved.

The society then unanimously elected to membership Drs. Sarah Millen, W. Liberty, Ia., and Dr. McCullough, Chicago.

Dr. E. P. Murdock then reported an interesting case, and presented the specimen of a fœtus, 7th mo., with the following history: The labor was a normal one, together with the presentation, the afterbirth being extremely small, and no liquor amnii.

Examination of the fœtus showed a double hare-lip, there being no articulation between the maxillary and inter-maxillary bones, and a deficient articulation of the ethmoid with the frontal, the occipital bone being absent, and the cerebellum lying outside the skull. No family history could be obtained of any consequence; the mother, an Irish Canadian, age 24, had enjoyed good health, had not been frightened in any way. Father, a Norwegian of good family history. Parents are usually able to assign some cause, imaginary or not, but not in this case. For years the subject of maternal impressions had been under discussion both among the laity and in the profession. Dr. Murdock, after many experiments upon eggs, painting, staining, varnishing, and placing them under peculiar conditions, had only negative results, and all authority was decisive that all such malformations as the one now shown are entirely independent of maternal impressions. The greatest number of abnormalities are found to occur in the brain and heart, especially in the lower orders of animals, where maternal impressions could not occur; sheep are frequently born headless, and monstrosities are of the most common occurrence in that family.

Among pigs the inter-maxillary bone is often deficient, and single-eyed pigs are very frequently seen.

In the human family duplex monsters are most frequent, and are never of opposite sex in any instance. Union of double monsters always extends to vital organs. In the Siamese twins the livers joined, and the abdominal cavities were directly connected.

In 1869, upon examination of Millie Christine, Dr. Murdock found the bodies joined at the tenth dorsal vertebra, the abdominal cavities united, one pelvic arch and four lower extremities; one set of arms, one vagina, and probably one uterus.

The proportion of sex in duplex monsters is largely the female, being four to one.

In monsters showing lack of development, that of the upper extremities is very common, and of the upper portion of the body in the median line.

In conclusion, Dr. Murdock spoke briefly of his great interest in the subject, and hoped to hear others speak of cases.

Dr. Campbell then reported a case which almost created a belief in maternal impressions, of a child born in normal labor, the pregnancy having also been normal, weighing at birth three and one half pounds, the head undeveloped, hair growing down to the eyes, which were closed, arms flexed, no hands, but rudimentary fingers on the wrists, toes webbed; it sucked and lived five months. The assigned cause of this montrosity as given by the mother was as follows: During the early part of pregnancy, while driving with her clergyman, who had a deformed arm similar in appearance to that of the monster, the horse ran away and frightened the lady, who sat rigid a long time, with arms flexed, watching the minister's hands.

Dr. Taggert thought that in all such cases no mother could tell where deformity would be; frights were of common occurrence in cities; marking must come in the ovum before impregnated, or in the spermatozoa.

Dr. Murdock then said that it was now established that the fœtus was wholly disconnected from the mother during gestation, and that the placenta was only a gland secreting vitalizing fluid. The child nurses through the umbilical cord in utero, and is no more liable to maternal impressions than a nursing child already born would be to the same impressions. There was no vascular connection between mother and child, no osmosis between the capillaries, but the placenta secretes fluid which nourishes the child. Shock and fright are afterthoughts, as stated by Dr. Taggert. Mothers almost universally expect deformity, or fear it, but such deformity is the rarest thing in the world. Nervous shocks may produce miscarriage, stopping development and throwing it off. There are no nerve filaments connecting child and mother. A lady had eight children, all deficient, and all had undeveloped bones in mesial line; all were still-born, or nearly so.

A man having had three wives all the children had extra thumbs, an excess of development.

Agassiz found in the embryo of fish a bifurcation which developed into two-tailed fish, an excess of development common in fishes. Their ova commonly show either deficient or excessive development, and in the spermatozoa of man deficiency of development has been noted by microscopic examination, but it would be impossible to prove that they would so develop.

Amalgamated races are more liable to deformity. In birds, while in their native state, no deformity can be found, and in 3600 specimens, prepared by the orator for the Smithsonian Institute, no deformity was observed, but in hybrid birds it is quite frequently met with, so much so that reproduction may become impossible.

Dr. Avery, in closing the discussion, expressed his interest in the specimen and cases reported. Peculiarities in the father, he had noticed, were frequently maintained among offspring. A gentleman in town has several boys, all of whom have very crooked little fingers, like the father, while the girls have not.

Dr. Murdock would inquire of the society in relation to certain cases seen lately in obstetric practice,—deep bilateral cervical lacerations. In one case, woman had pains forty-eight ours, examination showed no dilatation, but all subjective symptoms were present.

Dr. Avery asked his treatment in such cases. Reply, gave hypnotics, Battles' bromidia, viburnum, etc.

Dr. Taggert had one such case, lately, of bilateral laceration, in which false pains set in and stopped; he thought such cases were more apt to miscarry.

Dr. Murdock had not found laceration to interfere with impregnation; cervical catarrh, if present, should be relieved, then impregnation occurs; wished at some future time to have the subject brought up for consideration.

Dr. Avery thought that in very many cases of labor, false pains simulated impending labor.

Dr. Murdock replied that in all such cases he had found some injury, cervical, or otherwise.

Dr. Dobbin then reported a case with the following history: Last September was called to see a woman flowing freely, and had been, at intervals, for about seven months. The assigned cause was a fall, and a serous discharge preceded the hæmorrhage. On examination, found the os open enough to admit three fingers, and hæmorrhage free, with apparently some new growth in the cervical canal, though the examination was not a thorough one. After tamponing the vagina, the flowing ceased and the os was found nearly closed. The cervix was involved in a nodulated growth. There was no odor, but very intense pain. Emaciation was marked, but hæmorrhage remained slight. Dr. Byford, in consultation, advised ergot, and the hardened cervix seemed lately softer and smaller. The tumor surrounded the whole os, and was larger posteriorly. The body was normal in size.

Dr. Murdock thought the history indicated epithelioma; the odor being absent, did not exclude epithelioma.

Dr. Dobbin then stated that the case was so diagnosed by Dr. Byford, but thought the ergot was doing good.

Dr. Murdock thought that Dr. Byford was very enthusiastic on the subject of ergot; it might aid by cutting off the blood supply; he had found that iodized phenol gave the most relief from pain, iodine in carbolic acid, acting as a local anæsthetic.

A case seen at the Dispensary had the following history: First seen, October, 1882; examination revealed a large intra-mural fibroid and several fibromata. Length of canal, five and one-half inches. The case was also seen by Dr. Jackson. The woman had been treated nine years without benefit. A marked peculiarity of the case was that there had been no menorrhagia, but considerable pain. Treated by acetate of ammonium, varied by iodide of potassium and ammonium.

The general system was attended to by tonics, etc., and the tumor had gradually grown smaller, until at present about the size of an orange.

Present, about twelve members and several visitors. The society, on motion, adjourned.

J. H. TEBBETS, *Secretary.*

